# Pre-Intervention Assessment of *Toxocara* Infection in Dogs in Vietnam: A Community-Based Cross-Sectional Study

**DOI:** 10.3390/ani16091405

**Published:** 2026-05-03

**Authors:** Minh-Trang Thi Hoang, Dinh Ng-Nguyen, Ketsarin Kamyingkird, Van-Phuong Ngo, Tawin Inpankaew

**Affiliations:** 1 Department of Parasitology, Faculty of Veterinary Medicine, Kasetsart University, Lad Yao, Chatuchak, Bangkok 10900, Thailand; trangthiminh.h@ku.th (M.-T.T.H.); ketsarin.ka@ku.th (K.K.); 2 Department of Microbiology and Parasitology, Buon Ma Thuot Medical University, Buon Ma Thuot, Dak Lak 63100, Vietnam; 3 Graduate Program in Animal Health and Biomedical Sciences, Faculty of Veterinary Medicine, Kasetsart University, Bangkok 10900, Thailand; 4 Department of Veterinary Medicine, Faculty of Agriculture, Tay Nguyen University, Buon Ma Thuot, Dak Lak 63100, Vietnam; nndinh@ttn.edu.vn; 5 Department of Basic Sciences, Buon Ma Thuot Medical University, Buon Ma Thuot, Dak Lak 63100, Vietnam; nvphuong@bmu.edu.vn

**Keywords:** dogs, PCR, *Toxocara*, Vietnam

## Abstract

Dogs are key reservoirs of zoonotic infections, including *Toxocara canis*, a widely distributed parasite of major public health concern. In Vietnam, the parasite is highly prevalent in dog populations and humans. Epidemiological studies assessing infection and associated factors are essential to better understand transmission and to inform effective control strategies. We conducted a cross-sectional baseline survey to assess *Toxocara* infection in dogs in rural Vietnam. Fecal samples from 371 dogs were examined using centrifugal flotation (Sheather’s solution, specific gravity 1.2) and conventional polymerase chain reaction (PCR), alongside structured questionnaires on dog demographics and management. Using combined copromicroscopic and molecular methods, the overall prevalence of *Toxocara* infection was 44.7% (95% CI: 39.6–50.0). By microscopy alone, 29.9% (95% CI: 25.4–34.9) of samples were positive, while PCR detected *Toxocara* DNA in 41.2% (95% CI: 36.2–46.5) of dogs. Molecular analysis identified *T. canis* in 35.9% (95% CI: 31.0–41.0) and *T. cati* in 10.5% (95% CI: 7.7–14.2) of tested dogs. Dog age and deworming status were independently associated with PCR-detected *T. canis* infection. The elevated likelihood of infection among dogs that have never been dewormed highlights the importance of canine deworming. Questionnaire findings indicating suboptimal dog care and management highlight the need for community public health education to promote responsible ownership and reduce transmission risk. This baseline assessment provides essential evidence to inform targeted interventions and improve understanding of *Toxocara* transmission in endemic settings.

## Simple Summary

Parasitic infections that can pass from animals to humans remain an important but often overlooked health issue in many rural communities. One such parasite is *Toxocara canis*, which is commonly carried by dogs and can affect people through environmental contamination. This cross-sectional study was conducted to assess *Toxocara* infection in dogs in rural Vietnam. Fecal samples from 371 dogs were examined using copromicroscopic and molecular methods, and information was collected from their owners. The results showed that *Toxocara* infection is common, with molecular evidence of *T. canis* and *Toxocara cati*. Younger dogs and those that have not received anthelmintic treatment were more likely to be PCR-positive for *T. canis*. These findings highlight the role of basic animal care practices, particularly routine deworming and proper management, in controlling infection and reducing the risk to both animals and humans. The results provide practical information that can support community-based efforts to improve public health and reduce the burden of zoonotic diseases.

## 1. Introduction

Dogs are important reservoirs of zoonotic pathogens and play a significant role in the transmission of infections to humans. Among these, *Toxocara canis* is a globally distributed parasite of major public health importance, particularly in tropical and subtropical regions [1]. It is estimated that more than 100 million dogs worldwide have been infected with *Toxocara*, contributing to substantial environmental contamination through the shedding of parasite eggs [1]. These eggs can remain infective for prolonged periods under favorable environmental conditions, thereby sustaining transmission risk [2]. Adult dogs infected with *T. canis* typically exhibit mild or no clinical signs, while puppies are much more vulnerable, leading to gastrointestinal and respiratory disorders, poor growth, intestinal blockage, or even death [3]. In addition to its impact on dog health, *T. canis*, together with *T. cati* (the cat roundworm), is recognized as the principal causative agent of human toxocariasis [4]. Systematic reviews estimated that 19.0% of the world’s population has been infected or exposed to *Toxocara*, with human toxocariasis imposing an estimated annual burden of approximately 2.5 billion USD and 91,714 Disability-Adjusted Life Years (DALYs) lost among affected countries [5,6]. Transmission to humans is primarily via the accidental ingestion of infective *Toxocara* eggs and, less frequently, by ingesting infective larvae in the tissue of infected paratenic hosts [7]. Children and individuals with soil-exposed occupations, such as farming, gardening, and construction, are considered at the highest risk of *Toxocara* infection [7]. Human toxocariasis is usually asymptomatic or may result in several clinical symptoms, including visceral larva migrans (VLM), ocular larva migrans (OLM), covert toxocariasis (CT), and neural larva migrans (NLM) [7]. As dogs are the main definitive hosts of *T. canis*, control of infection in dogs is considered an important part of toxocariasis prevention. Regular anthelmintic treatment of dogs and public health education to improve owner awareness and hygiene practices have been proposed as practical strategies to reduce environmental contamination and zoonotic risk [8,9,10]. Currently, the effectiveness of these control measures in controlling *Toxocara* infection in dogs in community settings remains limited.

To detect infections in dogs, copromicroscopic methods are commonly used to identify *Toxocara* eggs due to their practicality and low cost. However, species-level identification of *Toxocara* eggs is challenging because of their morphological similarity under microscopic examination [11]. In contrast, polymerase chain reaction (PCR)-based methods can provide high sensitivity and accurate species-specific identification of *Toxocara* species [12,13].

*Toxocara* infection in dogs has been reported in many countries, particularly in countries lacking adequate veterinary care, uncontrolled dog populations, and absent control and preventive programs [1,14,15]. In Vietnam, *Toxocara* remains widely prevalent and is among the most common reported zoonotic parasites in dogs, with prevalence ranging from 29.7% to 37.7% [16,17,18,19,20]. Infected dogs may contribute to the maintenance of transmission through environmental contamination, thereby increasing the risk of human infection. In humans, serological surveys indicate that exposure to *Toxocara* remains high and widespread across the country [21,22,23]. Previous surveys in the country have largely relied on copromicroscopy to detect *Toxocara* eggs in dogs. Due to *Toxocara* species egg morphology being nearly identical [11], copromicroscopic results are typically reported as *Toxocara* or assumed to be *T. canis* in dogs. However, along with *T. canis* eggs, *T. cati* eggs have been found in dog feces in other countries [24,25,26]. Moreover, *T. malaysiensis*, confirmed in cats in Vietnam [27,28], might also contaminate dog feces. Therefore, copromicroscopy cannot determine the accurate prevalence of *T. canis* in dogs. To date, molecular data on *Toxocara* in Vietnam remain scarce, limiting understanding of species distribution in dogs and factors associated with *T. canis* infection, which are important for informing targeted control strategies.

This study aimed to assess *Toxocara* infection in dogs in a rural district of Dak Lak province, Vietnam, using combined copromicroscopic and molecular approaches to estimate overall and species-specific prevalence, assess infection intensity, and identify factors associated with PCR-detected *T. canis* infection.

## 2. Materials and Methods

### 2.1. Study Design and Sample Collection

A cross-sectional study was conducted between February and March 2025 in Krong Nang district, Dak Lak province, Vietnam. Krong Nang is located in the northern part of Dak Lak province and covers an area of approximately 615 square kilometers. The district has a tropical rainforest climate with two distinct seasons: a rainy season and a dry season. Administratively, Krong Nang comprised 12 commune-level administrative units.

In the Krong Nang district, eight hamlets were selected for baseline data collection on *Toxocara* infection in dogs. Four hamlets were randomly selected from a total of 14 hamlets in Eaho commune, and another four were randomly selected from 15 hamlets in Eatam commune. Eatam and Eaho communes were selected due to their comparable climatic conditions, socioeconomic characteristics, and dog ownership practices, facilitating meaningful comparison of epidemiological patterns. The data generated also serve as baseline information for a planned community-based intervention study.

A list of approximately 1600 households owning dogs in the selected hamlets was compiled with the assistance of local commune leaders, from which households were selected by simple random sampling using a computer-generated random number sequence. Dogs were enrolled upon owner consent, without restriction by sex, breed, or age. In households with one dog, that dog was sampled, whereas in households with two or more dogs, up to two dogs were randomly selected by assigning each dog a unique identifier and selecting individuals using simple random sampling. Approximately 5–10 g of feces were collected, homogenized in 5% *w*/*v* potassium dichromate, and stored at 4 °C in labeled containers until analysis. In addition, dog owners completed a structured questionnaire capturing household dog numbers; dog characteristics (age, sex, and breed); practices of caring for and managing dogs of the owner; and the deworming history of each dog. Each dog was assigned a unique code linking their fecal sample and questionnaire data.

The required sample size (*n* = 358) was calculated using a single proportion population formula (1), assuming an estimated prevalence (*p*) of *T. canis* infection of 35.7% (data derived from a pilot study, *n* = 126), a margin of error (*d*) of 0.05, and a 95% confidence level (Z1−α22 = 1.96). Therefore, the total number of samples was increased (*n* = 371) to ensure sufficient statistical power and to account for potential sample attrition.

(1) n=[Z1−α22p(1−p)]/d2

### 2.2. Copromicroscopic Examination

The fecal samples were examined for the presence of *Toxocara* spp. eggs and for egg counts (expressed as eggs per gram, EPG) using a fecal flotation technique with Sheather’s solution (specific gravity of 1.2 g/cm^3^) as the flotation solution [29]. Briefly, 2 g of each fecal sample was suspended in distilled water at a 1:4 ratio to create a homogenized mixture. The suspension was strained and transferred into a 10 mL centrifuge tube, then centrifuged at 3000× *g* for 2 min. After discarding the supernatant, the remaining pellet was mixed with Sheather’s solution. The tube was filled with additional Sheather’s solution to form a convex meniscus, and a coverslip was placed on top. After 10 min, the coverslip was then transferred to a microscope slide and examined for the presence of eggs of *Toxocara* species under light microscopy at 10× and 40× magnification using a zig-zag pattern. *Toxocara* eggs were counted across the entire coverslip, and the final count was multiplied by four to determine the EPG based on the proportion of fecal material represented on the examined coverslip. All slides were examined by trained examiners using standardized procedures, and a subset of samples was re-examined by an additional examiner to ensure consistency. Examiners were blinded to PCR results and questionnaire data.

### 2.3. DNA Extraction and Molecular Analysis

All fecal samples (200 mg each) were extracted for genomic DNA using an E.Z.N.A.^®^ Stool DNA kit (Omega Bio-tek Inc., Norcross, GA, USA) according to the manufacturer’s instructions with a minor modification. At the first step, fecal homogenization was performed using a Buller Blender Lite machine (Next Advance, Inc., Troy, NY, USA) to enhance the disruption of *Toxocara* spp. eggs. Final DNA samples were stored at −20 °C, ready for PCR analysis. Conventional PCR (cPCR) was used to detect *T. canis*, *T. cati*, and *T. malaysiensis* DNA using the species-specific primer sets targeting the internal transcribed spacer (ITS) region of ribosomal DNA, with each species amplified in separate reactions (Table 1). The presence of target DNA was determined based on the expected amplicon size for each species (Table 1).

The amplification reactions were carried out in a final volume of 20 μL containing 4 μL of PCR Master Mix (HOT FIREPol^®^ Blend Master Mix, Solis BioDyne, Tartu, Estonia), 1 μL (10 pmol) of each primer, 2 μL of DNA template, and 12 μL of double-distilled water. The PCR conditions consisted of an initial denaturation at 95 °C for 15 min, followed by 35 amplification cycles of a denaturation step at 95 °C for 30 s, annealing at 58 °C for 30 s, and extension at 72 °C for 30 s, and a final extension at 72 °C for 10 min. To minimize the risk of cross-contamination, DNA extraction, PCR setup, and post-amplification analysis were conducted in separate areas. Positive controls consisting of previously confirmed DNA samples for each target species and negative controls (no-template controls) were included in every PCR run. Electrophoresis was performed by loading PCR products onto a 1.5% (*w*/*w*) agarose gel containing Prime Juice Preloading Fluorescent Stain (Bio-Helix Co., Ltd., Taipei, Taiwan), and bands were then visualized using an ultraviolet transilluminator.

### 2.4. DNA Sequencing and Phylogenetic Analysis

Five PCR-positive samples identified as *T. canis* and five as *T. cati* were randomly selected for sequencing using Sanger sequencing by U2Bio (Seoul, Republic of Korea). The obtained sequences were compared to other sequences in Genbank using the BLASTn tool via the NCBI web interface (https://blast.ncbi.nlm.nih.gov/Blast.cgi (accessed on 25 December 2025)) after visualizing by Finch TV version 1.4.0 trace viewer (Geospiza, Inc., Seattle, WA, USA). Multiple sequence alignment of ITS nucleotide sequences from this study and reference *Toxocara* spp. sequences downloaded from Genbank were conducted using the BioEdit program (version 7.7). Phylogenetic analyses were then constructed using Mega 12 software with the neighbor-joining analyses conducted using Tamura-Nei parameter distance estimates on a 327-base pair region of the aligned sequences. Bootstrap analyses were conducted using 1000 replicates.

### 2.5. Statistical Analysis

Two diagnostic methods were used in this study (copromicroscopy and PCR). Two outcome variables were defined for analysis: (i) *Toxocara* egg detection by copromicroscopy and (ii) *Toxocara* DNA detection by PCR (including *T. canis* and/or *T. cati*). In addition, an overall prevalence was calculated as the proportion of dogs positive by either copromicroscopy or PCR results. For each outcome, prevalence was calculated using the total number of dogs examined as the denominator.

All data analyses were performed using R Statistical Software version 4.4.3 [31]. The prevalence and their 95% confidence intervals (CI) were calculated for categorical data. Geometric mean (GM) egg count (EPG) and their respective 95% CI were calculated to describe infection intensity. Agreement between copromicroscopy and PCR was assessed using Cohen’s kappa statistic, with PCR positivity defined as the detection of *T. canis* and/or *T. cati* DNA.

A mixed-effects logistic regression model was used to identify factors associated with PCR-detected *T. canis* infection in dogs. In this model, individual dogs testing positive for *T. canis* DNA by PCR were assigned a value of 1, or 0 otherwise, as the outcome variable. Unconditional associations between each explanatory variable derived from the questionnaire data and the outcome variable were first assessed using the odds ratios and either the chi-square test or Fisher’s exact test. Explanatory variables with unconditional associations at a two-sided *p* < 0.2 level were selected for inclusion in the multivariable analysis as a screening approach to avoid excluding potentially relevant factors at an early stage. Multivariable logistic regression analysis was then performed using an iterative backward elimination procedure. Explanatory variables were sequentially removed from the model, beginning with the least statistically significant, until the estimated regression coefficients for the retained explanatory variables were significant at *p* < 0.05. Explanatory variables that were excluded at the initial screening stage were subsequently re-evaluated for inclusion in the final model and were retained if their inclusion changed any of the estimated regression coefficients by more than 20%. Multicollinearity among explanatory variables included in the multivariable model was assessed using variance inflation factors (VIF). Biologically plausible two-way interactions were tested, and none were statistically significant at *p* < 0.05. To account for the hierarchical structure of the data (dogs nested within households and hamlets), household was included as a random intercept in the mixed-effects model. A hamlet-level random effect was explored but not retained due to negligible variance and the limited number of hamlets, which may lead to unstable estimates.

## 3. Results

### 3.1. General Characteristics of the Dog Population

A total of 371 dogs from 242 households were included in the study. The characteristics of the study population are summarized in Table 2. The number of dogs sampled was similar between sexes. Adult dogs constituted the largest proportion of the study population (59.8%), followed by puppies (28.8%) and juveniles (11.3%). Most dogs were of local breed (88.1%), while mixed and foreign breeds accounted for 6.2% and 5.7%, respectively.

A large proportion of dogs were allowed to roam freely (75.2%) and had frequent contact with other animals (72.0%). Notably, 78.2% of dogs were allowed to defecate within the household area, but only 15.9% of the feces were treated by owners. The percentage of dogs that received deworming and a bath was low at 24.5% and 15.4%, respectively. The number of dogs kept per household ranged from one to 11.

### 3.2. Copromicroscopic Results and Egg Intensity by Age Group of Dogs

Of the 371 fecal samples examined, *Toxocara* spp. eggs were detected in 111 samples, accounting for 29.9% (95% CI: 25.4–34.9). The prevalence of *Toxocara* eggs detected was highest in puppies (49.5%, 53/107), slightly lower in juveniles (47.6%, 20/42), and adults had the lowest prevalence (17.1%, 38/222).

**Figure 1:**
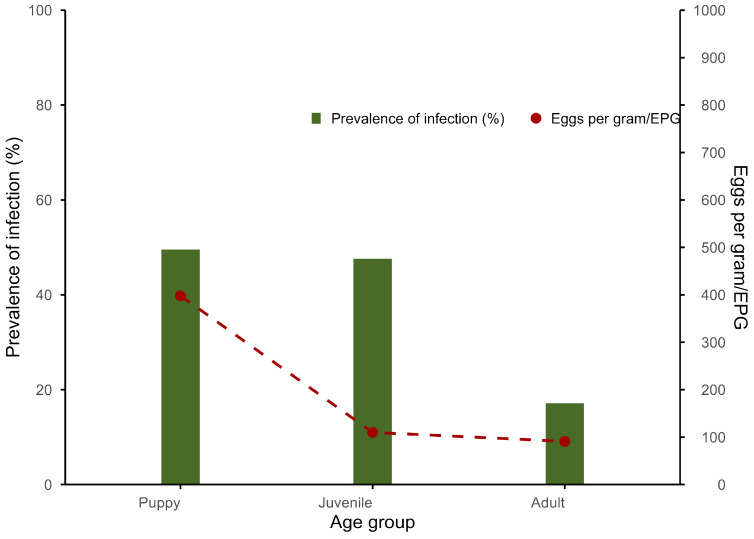
Prevalence and intensity (EPG) of *Toxocara* spp. eggs detected in dogs according to age group by copromicroscopy.

The GM infection intensity of *Toxocara* spp. eggs was 191 EPG (95% CI: 129–283). *Toxocara* infection intensities were similar in adults and juveniles but were higher in puppies (Figure 1).

### 3.3. Molecular Detection

With the PCR method, *Toxocara* DNA was detected in 41.2% (153/371, 95% CI: 36.2–46.5) of dogs. Among the samples, 13 samples were positive by microscopy but negative by PCR, whereas 55 were PCR-positive but microscopy-negative. When results from both methods were combined, the overall prevalence of *Toxocara* infection was 44.7% (166/371, 95% CI: 39.6–50.0). Agreement between copromicroscopy and PCR was substantial, with a Cohen’s kappa value of 0.61 (*p* < 0.001).

Of the 153 samples positive for *Toxocara* DNA, *T. canis* and *T. cati* DNA were detected in 133 samples (35.9%, 95% CI: 31.0–41.0) and 39 samples (10.5%, 95% CI: 7.7–14.2), respectively. Co-detection of *T. canis* and *T. cati* DNA was observed in 19 samples (5.1%, 95% CI: 3.2–8.0). No *T. malaysiensis* DNA was detected in the examined samples.

### 3.4. Phylogenetic Analysis

The phylogenetic analysis showed that all *T. canis* and *T. cati* isolates obtained in this study clustered within their respective species-specific clades together with reference sequences from GenBank. The corresponding phylogenetic tree and detailed sequence comparisons are provided in Supplementary Figure S1.

### 3.5. Factors Associated with PCR-Detected T. canis Infection

Univariate analysis identified several factors associated with PCR-detected *T. canis* infection (Table 3). Variables with *p* < 0.2 were included in the mixed-effects logistic regression model.

The mixed-effects logistic regression analysis identified dog age and deworming status as factors associated with PCR-detected *T. canis* infection (Table 4). The final model included 371 observations, and a random intercept for household was included to account for clustering of dogs within households. Puppies (adjusted OR = 3.03, 95% CI: 1.81–5.10) and juveniles (adjusted OR = 2.02, 95% CI: 1.01–4.05) had higher odds of infection compared with adult dogs. Dogs that had not been dewormed were 1.75 (95% CI: 1.00–3.07) times more likely to be PCR-positive for *T. canis* than those that had been dewormed. No evidence of multicollinearity was observed.

## 4. Discussion

The overall prevalence of *Toxocara* infection was 44.7% based on combined copromicroscopic and molecular methods, indicating a substantial level of infection in the study area. Estimates based on microscopy (29.9%) and PCR (41.2%) differed, reflecting differences in diagnostic sensitivity. The inclusion of molecular methods enabled species-specific detection and improved the characterization of *Toxocara* infection in dogs in Vietnam. The high overall prevalence observed in this study may be influenced by a tropical climate, which provides appropriate temperature and moisture for the survival of parasite eggs. In addition, the dog population at the study site is largely unmanaged, especially with a high proportion of free-roaming dogs (75.2%), and most had not received anthelmintic treatment (75.5%). Furthermore, 78.2% of dogs were allowed to defecate within household areas, but only 15.9% of feces were properly removed. These conditions may collectively contribute to the high *Toxocara* infection in the study dog population.

By employing conventional PCR, *T. canis* DNA was detected in 35.9% (95% CI: 31.0–41.0) of the dogs, as confirmed by DNA sequencing. Compared with reports from other countries using molecular detection methods, the prevalence observed in our study was considerably higher. In Thailand, a PCR-based study found a prevalence of 5.4% in stray dogs, lower than that found in our study [32]. Similarly, lower prevalence was also recorded in Cambodia at 15.0% [33] and the USA at 22.2% [34], Iran at 10.4% [35], and Iraq at 26.0% [36]. Besides *T. canis*, DNA of *T. cati* was detected in 39/371 samples. This finding is consistent with previous studies indicating that dogs shed eggs of both *T. canis* and *T. cati* [24,25,26]. Although true host switching of *Toxocara* spp. might be an exceptional event, an experimental study failed to produce patent infection of *T. cati* in dogs [26]. In addition, examination of adult worms collected from naturally infected dogs in Vietnam has confirmed *T. canis* as the sole species identified [16,18,27]. Therefore, the detection of *T. cati* DNA in dog feces may be associated with several factors, including coprophagy, a common behavior in dogs [26], as well as environmental contamination, mixed fecal samples, or potential laboratory cross-contamination. Although reported in cats in Vietnam [27,28], no *T. malaysiensis* egg contamination was detected in the examined dog feces.

Our results showed a lower prevalence of *Toxocara* eggs detected by centrifugal flotation (29.9%) compared with PCR-based detection of *Toxocara* DNA (41.2%). Although PCR detected a higher prevalence, agreement between the two methods was substantial, indicating good concordance despite differences in diagnostic principles. PCR may detect low-intensity infections or degraded eggs that are not visible by microscopy, whereas copromicroscopic methods rely on the presence of intact eggs and may therefore underestimate overall prevalence. In addition, 13 samples were positive by microscopy but negative by PCR, which may be explained by uneven egg distribution, low DNA yield, or the presence of PCR inhibitors in feces. Molecular analysis also provides a reliable method for species identification. Differentiation of *Toxocara* eggs at the species level is essential for generating accurate epidemiological data and for informing effective control and prevention strategies against toxocariasis in humans and animals. *Toxocara* eggs are easy to recognize under microscopic examination, but differentiation between species is challenging. Several studies have identified the differences in size and shell surface between *T. canis* and *T. cati*, but these characteristics are not consistent and overlap [11,26]. As a result, copromicroscopic surveys in dogs often report detected eggs in the genus *Toxocara* or presume solely *T. canis*. Yet *T. cati* has also been detected in dog feces, indicating that assigning all canis-derived eggs to *T. canis* can lead to overestimated prevalence, especially in areas where *T. cati* is common. Furthermore, while microscopy remains a useful and cost-effective diagnostic method, it becomes laborious and time-consuming for large sample sets; molecular detection, in contrast, is less labor-intensive as sample size increases [24]. Therefore, molecular approaches are a useful method for studies addressing *Toxocara* prevalence, particularly where species differentiation of *Toxocara* eggs at the species level is essential.

The results of the mixed-effects logistic regression analysis indicated that dog age and deworming status were significantly associated with PCR-detected *T. canis* infection in dogs. Adult dogs exhibited significantly lower odds of being PCR-positive compared to puppies and juveniles (Table 4), similar to the results of Ng-Nguyen and Nguyen (2024) and Yang et al. (2025) [19,37]. This age-related pattern is biologically plausible, as puppy and juvenile dogs are more susceptible to *T. canis* infection due to vertical transmission routes, including transplacental and transmammary transmission, as well as the immaturity of their immune systems [10,38]. Dogs that had not been dewormed showed higher odds of PCR-detected *T. canis* infection (adjusted OR = 1.75, 95% CI: 1.00–3.07) compared with those that received anthelmintic treatment. Our result is consistent with previous studies demonstrating an association between deworming and *T. canis* infection [37,39]. Implementing regular deworming is the most effective strategy to minimize *T. canis* infections in dogs. Based on our findings, anthelmintic treatment should be administered to dogs of all ages to effectively reduce environmental contamination and limit exposure for humans and animals. As the highest-risk population for *T. canis* infection, young dogs require intensive and frequent anthelmintic treatment to interrupt early-life transmission. Although the prevalence of patent infection decreased with increasing age, *T. canis* infection in adult dogs should not be overlooked. As shown in our results and supported by several surveys, *T. canis* in adult dogs has been detected at relatively high prevalence, indicating that adult dogs still play a significant role in the transmission [19,33,40]. Furthermore, administering appropriate anthelmintic regimens to pregnant bitches helps to reduce vertical transmission, which is the main route of *T. canis* infection in puppies [10,41].

In Vietnam, serological studies have consistently reported substantial *Toxocara* seropositivity in humans, indicating widespread and sustained exposure to the parasite [21,22,23]. Dogs play an important role in maintaining transmission by continuously shedding large quantities of parasite eggs that can remain viable and infectious in the environment for long periods [1,2]. Humans typically become infected through contact with contaminated environments or infected hosts [7]. The high prevalence of *Toxocara* egg shedding in dogs found in the study and previous studies in Vietnam suggests a potential role of dogs as a reservoir for environmental contamination and human exposure [16,17,18,19]. Environmental and behavioral factors, including climate, sanitation practices, and seasonal variation, may further influence the persistence and transmission of *Toxocara* eggs in the environment; however, these factors were not comprehensively assessed in the present study. These findings emphasize the need for targeted control measures based on local epidemiological evidence to effectively reduce the parasite burden in both humans and animals in endemic regions, including Vietnam.

This study has several limitations. First, the study was conducted in two purposively selected communes within a single district, which may limit the representativeness of the findings and introduce selection bias, thereby restricting generalizability to other regions. Second, households were randomly selected from a list of dog-owning households compiled and verified with the assistance of local authorities; however, the list may not have been fully comprehensive, and some selection bias cannot be excluded. Third, the cross-sectional design and limited sampling period may not capture seasonal variation in *Toxocara* transmission. Despite these limitations, the study benefits from the use of both copromicroscopic and molecular methods to improve detection accuracy and provides baseline data to inform future intervention studies. Further studies incorporating broader geographic coverage and longitudinal designs are needed to better understand transmission dynamics in endemic regions, including Vietnam.

## 5. Conclusions

This baseline survey provides the first molecular investigation of *Toxocara* egg shedding in dogs in Vietnam. The overall prevalence of infection was 44.7% based on combined copromicroscopic and molecular methods, indicating a substantial level of infection in the study area. PCR-based analysis identified *T. canis* in a considerable proportion of dogs, with dog age and deworming status identified as key factors associated with PCR-detected *T. canis* infection, while environmental conditions may also influence transmission dynamics. These findings serve as important baseline data for evaluating community-based interventions aimed at reducing zoonotic transmission and highlight the need for locally appropriate, dog-focused control measures to reduce environmental contamination and transmission risks for both humans and animals.

## Figures and Tables

**Table 1:** Details of oligonucleotide primer sets used in this study for the detection of *Toxocara* species.

Target Species	Primers	Oligonucleotide Sequence 5′–3′	Size (bp)	References
*T. canis*	Tcan-F	5′-AGTATGATGGGCGCGCCAAT-3′	380	[13]
	NC2-R	5′-TAGTTTCTTTTCCTCCGCT-3′		
*T. cati*	Tcat-F	5′-GGAGAAGTAAGATCGTGGCACGCGT-3′	370	[13]
	NC2-R	5′-TAGTTTCTTTTCCTCCGCT-3′		
*T. malaysiensis*	Tma-F	5′-ACACAACACAATCTGTTACA-3′	700	[30]
	NC2-R	5′-TAGTTTCTTTTCCTCCGCT-3′		

**Table 2:** General characteristics of 371 dogs in the study.

Variables	Number of Individuals	Percentage	95% CI *
Total number of dogs	371		
Residence place:			
*Eatam*	196	47.2	42.0–52.4
*Eaho*	175	52.8	47.6–58.0
Sex:			
*Male*	162	43.7	38.6–48.9
*Female*	209	56.3	51.1–61.4
Age:			
*Adult*	222	59.8	54.6–64.8
*Juvenile*	42	11.3	8.4–15.1
*Puppy*	107	28.8	24.3–33.8
Breed of dog:			
*Local*	327	88.1	84.3–91.2
*Mixed breed*	23	6.2	4.1–9.3
*Foreign*	21	5.7	3.6–8.7
Allowed to roam freely:			
*Yes*	279	75.2	70.4–79.5
*No*	92	24.8	20.6–29.6
Defecation area:			
*Inside the home area*	290	78.2	73.5–82.2
*Outside the home area*	81	21.8	17.8–26.5
Dewormed:			
*Yes*	91	24.5	20.3–29.3
*Never*	280	75.5	70.7–79.7
Bathed:			
*Yes*	57	15.4	11.5–19.5
*No*	314	84.6	80.5–88.1
Dog feces management:			
*Yes*	59	15.9	12.4–20.1
*No*	312	84.1	79.9–87.6
Contact with other animals:			
*Yes*	267	72.0	67.1–76.4
*No*	104	28.0	23.6–33.0
Dogs per household:			
*1*	30	8.1	5.6–11.5
*2*	110	29.7	25.1–34.6
*3*	68	18.3	14.6–22.7
*>3*	163	43.9	38.8–49.2

* confidence interval.

**Table 3:** Univariate analysis of factors associated with PCR-detected *T. canis* infection in dogs.

Variables	Number Positive (%)	Number of Individuals	Crude OR (95% CI *)	*p*-Value
Total number of dogs	133 (35.9)	371		
Residence place:				
*Eatam*	72 (36.7)	196	1.00	0.707
*Eaho*	61 (34.9)	175	0.92 (0.60–1.41)	
Sex:				
*Male*	64 (39.5)	162	1.00	0.196
*Female*	69 (33.0)	209	0.76 (0.49–1.16)	
Age:				
*Adult*	58 (26.1)	222	1.00	0.000
*Juvenile*	18 (42.9)	42	2.12 (1.06–4.18)	
*Puppy*	57 (53.3)	107	3.22 (1.99–5.25)	
Breed of dog:				
*Foreign*	5 (23.8)	21	1.00	0.384
*Local*	118 (36.1)	327	1.81 (0.69–5.64)	
*Mixed breed*	10 (43.5)	23	2.46 (0.69–9.65)	
Allowed to roam freely:				
*No*	28 (30.4)	92	1.00	0.212
*Yes*	105 (37.6)	279	1.38 (0.84–2.31)	
Defecation area:				
*Inside the home area*	104 (35.9)	290	1.00	0.992
*Outside the home area*	29 (35.8)	81	0.99 (0.59–1.66)	
Dewormed:				
*Yes*	22 (24.2)	91	1.00	0.008
*Never*	111 (39.6)	280	2.05 (1.22–3.58)	
Bathed:				
*Yes*	19 (33.3)	57	1.00	0.667
*No*	114 (36.3)	314	1.14 (0.63–2.11)	
Dog feces management:				
*Yes*	15 (25.4)	59	1.00	0.068
*No*	118 (37.8)	312	1.78 (0.97–3.44)	
Contact with other animals:				
*No*	32 (30.8)	104	1.00	0.203
*Yes*	101 (37.8)	267	1.37 (0.85–2.24)	
Dogs per household:				
*1*	9 (30.0)	30	1.00	0.473
*2*	40 (36.4)	110	1.33 (0.57–3.32)	
*3*	20 (29.4)	68	0.97 (0.39–2.57)	
*>3*	62 (38.0)	163	1.51 (0.67–3.66)	

* confidence interval.

**Table 4:** Estimated regression coefficients from a mixed-effects logistic regression model of factors associated with PCR-detected *T. canis* infection in dogs.

Explanatory Variable	Number Positive	Number of Individuals	Regression Coefficient (SE)	*p*-Value	Adjusted OR (95% CI *)
Intercept	133	371	−1.4543 (0.2794)	0.000	
Age:					
*Adult*	58	222	Reference		1.00
*Juvenile*	18	42	0.7041 (0.3539)	0.047	2.02 (1.01–4.05)
*Puppy*	57	107	1.1100 (0.2645)	0.000	3.03 (1.81–5.10)
Dewormed:					
*Yes*	22	91	Reference		1.00
*Never*	111	280	0.5609 (0.2858)	0.049	1.75 (1.00–3.07)

* confidence interval.

## Data Availability

The data presented in this study are available on request from the corresponding author.

## Supplementary Materials

The following supporting information can be downloaded at https://www.mdpi.com/article/10.3390/ani16091405/s1, Figure S1: Phylogenetic relationships among *Toxocara* based on ITS-2 sequence data inferred using the Neighbor-Joining method. *Toxascaris leonina* was used as the outgroup. Sequences obtained from dogs in this study are indicated by blue circles for *T. canis* and blue triangles for *T. cati*.

## Abbreviations

The following abbreviations are used in this manuscript:

PCR	Polymerase chain reaction
CI	Confidence interval
DALYs	Disability-Adjusted Life Years
EPG	Eggs per gram
ITS	Internal transcribed spacer
VLM	Visceral larva migrans
OLM	Ocular larva migrans
CT	Covert toxocariasis
NLM	Neural larva migrans
NGS	Next-generation sequencing
GM	Geometric mean
