# Supplementary material for: Pre-Intervention Assessment of Toxocara Infection in Dogs in Vietnam: A Community-Based Cross-Sectional Study
Source: Animals (Basel). 2026 May 3;16(9):1405. doi: 10.3390/ani16091405 (PMC13162858; doi:10.3390/ani16091405)
Supplement: Supplementary file 1 [file animals-16-01405-s001.zip › animals-4263979-supplementary.pdf]

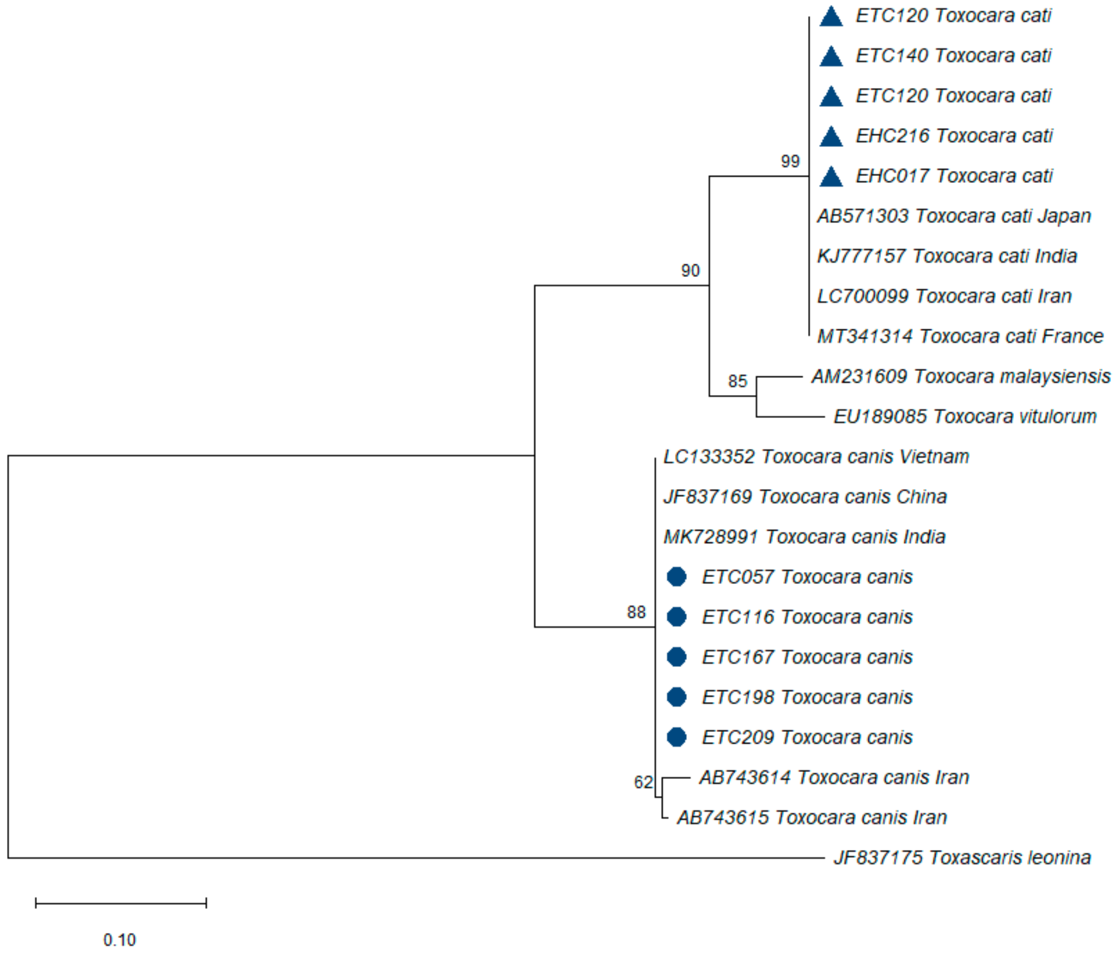

Figure S1. Phylogenetic relationships among *Toxocara* based on ITS-2 sequence data inferred using the Neighbor-Joining method. *Toxascaris leonina* was used as the outgroup. Sequences obtained from dogs in this study are indicated by blue circle for *T. canis* and blue triangles for *T. cati*.
